# Early toxicity and patient reported quality-of-life in patients receiving proton therapy for localized prostate cancer: a single institutional review of prospectively recorded outcomes

**DOI:** 10.1186/s13014-018-1127-6

**Published:** 2018-09-17

**Authors:** Howard J. Lee, Meghan W. Macomber, Matthew B. Spraker, Stephen R. Bowen, Daniel S. Hippe, Angela Fung, Kenneth J. Russell, George E. Laramore, Ramesh Rengan, Jay Liao, Smith Apisarnthanarax, Jing Zeng

**Affiliations:** 1 Duke University School of Medicine, 10 Duke Medicine Circle, Durham, NC 27710 USA; 2 Department of Radiation Oncology, University of Washington School of Medicine, 1959 NE Pacific Street, Campus, Box 356043, Seattle, WA 98195 USA; 3 Department of Radiology, University of Washington School of Medicine, 1959 NE Pacific Street, Campus, Box 357115, Seattle, WA 98195 USA; 4 Seattle Cancer Care Alliance Proton Therapy Center, 1570 N 115th St, Seattle, WA 98115 USA

**Keywords:** Prostate cancer, Proton therapy, Quality of life, Patient reported outcomes

## Abstract

**Background:**

We report prospectively captured clinical toxicity and patient reported outcomes in a single institutional cohort of patients treated for prostate cancer with proton beam therapy (PBT). This is the largest reported series of patients treated mostly with pencil beam scanning PBT.

**Methods:**

We reviewed 231 patients treated on an IRB approved institutional registry from 2013 to 2016; final analysis included 192 patients with > 1-year of follow-up. Toxicity incidence was prospectively captured and scored using CTCAE v4.0. International Prostate Symptoms Score (IPSS), Sexual Health Inventory for Men (SHIM) score, and Expanded Prostate Cancer Index Composite (EPIC) bowel domain questionnaires were collected at each visit. Univariate Cox regression was used to explore associations of grade 2+ toxicity with clinical, treatment, and dosimetric variables.

**Results:**

Median follow-up was 1.7 years. Grade 3 toxicity was seen in 5/192 patients. No grade 4 or 5 toxicity was seen. Patient reported quality-of-life showed no change in urinary function post-radiation by IPSS scores. Median SHIM scores declined by 3.7 points at 1-year post-treatment without further decrease beyond year 1. On univariate analysis, only younger age (HR = 0.61, *p* = 0.022) was associated with decreased sexual toxicity. EPIC bowel domain scores declined from 96 at baseline (median) by an average of 5.4 points at 1-year post-treatment (95% CI: 2.5–8.2 points, *p* < 0.001), with no further decrease over time. Bowel toxicity was mostly in the form of transient rectal bleeding and was associated with anticoagulation use (HR = 3.45, *p* = 0.002).

**Conclusions:**

Grade 3 or higher toxicity was rare at 2-years after treatment with PBT for localized prostate cancer. Longer follow-up is needed to further characterize late toxicity and biochemical control.

**Trial registration:**

NCT, NCT01255748. Registered 1 January 2013.

## Background

Numerous treatment options exist for localized prostate cancer. External beam radiation therapy is a non-invasive option that has shown similar disease control rates compared to other approaches such as radical prostatectomy and brachytherapy [1–5], and treatment modality choice is generally a consideration of side effect profile and shared decision making.

Dose escalation has been shown to improve cancer control in prostate cancer [6]. Delivering the high radiotherapy doses to the prostate necessary to improve outcomes can be challenging due to tolerance of surrounding organs at risk, in particular rectum and bladder. Higher radiation doses using conventional external beam techniques have been shown in several retrospective studies to increase the risk of late treatment related toxicity [7, 8]. Proton beam therapy (PBT) has emerged as an external beam radiotherapy treatment option. The unique dosimetric characteristics of PBT allow for dose escalation while reducing dose to surrounding structures, especially in the low-dose bath [7].

While numerous studies have demonstrated this reduction in low dose bath to surrounding normal tissues such as rectum and bladder, the clinical advantage remains unclear [8]. Only a few centers have reported clinical outcomes of patients treated with PBT for prostate cancer [9–18]. Existing proton literature in prostate cancer is mostly based on passive scattering proton technology, while pencil beam scanning (PBS) has some dosimetric advantages [19]. We present our 2-year outcomes regarding toxicity and patient reported quality of life (QOL) for patients receiving PBT at our institution. To our knowledge, this is the largest reported series of patients treated mostly with PBS proton technology.

## Methods

### Patient details and data collection

We reviewed 231 patients treated consecutively at our institution with PBT for localized prostate cancer on a prospective IRB-approved registry. Patients were excluded for prior radical prostatectomy, prior radiotherapy, treatment with mixed photon and proton radiation, or follow-up < 1-year. Total 192 patients were included in this analysis. Baseline characteristics are outlined in Table 1. All patients underwent institutional pathology review of prostate biopsy to confirm Gleason score. Intermediate risk patients by D’Amico risk groups underwent computed tomography (CT) and/or magnetic resonance imaging (MRI) of the pelvis. Patients with high-risk disease also had technetium bone scans.

**Table 1:** Patient characteristics

Variable	No. (%) or Median (Range)
Age, years	68 (50–85)
Race and ethnicity	
African-American	3 (1.6%)
Asian	4 (2.1%)
Hispanic	2 (1.0%)
White	176 (91.7%)
Unknown	7 (3.6%)
T stage	
T1	104 (54.2%)
T2a	49 (25.5%)
T2b	25 (13.0%)
T2c	4 (2.1%)
T3-T4	10 (5.2%)
PSA, ng/ml	7.2 (1.6–69.6)
Gleason score	
6	42 (21.9%)
7 = 3 + 4	80 (41.7%)
7 = 4 + 3	36 (18.8%)
8	14 (7.3%)
9–10	20 (10.4%)
Risk category	
Low	38 (19.8%)
Intermediate	104 (54.2%)
High	50 (26.0%)
Baseline IPSS Bother score^a^	2 (0–7)
Baseline IPSS score^a^	6 (0–28)
Baseline EPIC bowel domain score^a^	96 (61–100)
Baseline SHIM score^ab^	18 (0–25)
Comorbidities	
History of diabetes	19 (9.9%)
History of hypertension	96 (50.0%)
History of inflammatory bowel disease	2 (1.0)
History of hemorrhoids	26 (13.5%)
Smoking status^a^	
Never	103 (57.5%)
Former	65 (36.3%)
Current	11 (6.1%)
Aspirin use	74 (38.5%)
Anticoagulant use	22 (11.5%)
Pre-treatment urologic function	
Alpha blocker use	37 (19.3%)
Alpha reductase inhibitor use	14 (7.3%)
TURP	9 (4.7%)
Androgen deprivation therapy	71 (37.0%)
Low risk	1/71 (1.4%)
Intermediate risk	27/71 (38.0%)
High risk	43/71 (60.6%)
Pencil beam vs. uniform scanning	144 vs 48 (75.0% vs 25%)
Number of fields/day	
1	92 (48%)
2	100 (52%)
Whole pelvis radiation	19 (9.9%)
US-based prostate volume, cm^3^	40 (12–100)

^a^Those with missing values were excluded from the corresponding summary: IPSS bother score (*n* = 7), IPSS score (*n* = 4), EPIC score (*n* = 28), SHIM score (*n* = 14), smoking status (*n* = 13), and US-based prostate volume (*n* = 12);

^b^Based on patients not receiving androgen deprivation therapy (*n* = 121)

### Outcome and follow up

All patients were evaluated at pre-treatment, weekly on-treatment, and every 3–4 months for the first year post-treatment, then at 6-month intervals. Acute toxicity was defined as from start of radiation therapy to 90 days after treatment completion, and late toxicity was defined as at any time after 90 days. Patients prospectively completed International Prostate Symptoms Score (IPSS), Sexual Health Inventory for Men (SHIM) score, and Expanded Prostate Cancer Index Composite (EPIC) bowel domain questionnaires. Toxicity was scored using Common Terminology Criteria for Adverse Events, version 4.0 (CTCAE).

### Treatment details

Proton therapy treatment included CT simulation at 1.25 mm slice thickness in the supine position using vacuum-locked body mold for immobilization, with instructions for full bladder and empty rectum. All patients had placement of three Visicoil fiducial markers under transrectal ultrasound guidance (IBA Dosimetry GmbH, Schwarzenbruck, Germany). All patients were treated with daily rectal balloons filled with 90 cc of saline. Patients were also instructed to drink 16 oz. of water 30 min to one hour prior to treatment to ensure a full bladder, with adjustments made based on individual urinary function. Treatment planning was categorized according to whether the clinical target volume (CTV) included prostate only, prostate and seminal vesicles, or inclusion of pelvic nodes for high risk disease at the discretion of the treating physician. Planning target volume (PTV) was an expansion of 5 mm in all directions except 4 mm posteriorly. Target coverage goal was 95% of PTV receiving 100% of prescribed dose, and 100% of PTV receiving ≥95% of prescribed dose. All patients received treatment with standard fractionation (1.8–2.0 CGE fractions). Prescribed dose was 79.2–81 CGE in 85% of patients, and 75.6–81 CGE in > 95% of patients. Prescribed doses to pelvic lymph nodes were 45–50.4 CGE. Maximum dose was kept to < 103% of prescribed dose. Organs at risk (OAR) doses are show in Table 2.

**Table 2:** Patient dosimetry (*N* = 192)

Variable	No. (%) or Median (Range)
Dose	
< 79.2 Gy (RBE)	27 (14.1%)
≥ 79.2 Gy (RBE)	165 (85.9%)
DVH Parameters	
Rectal wall V50, %	30.7 (8.0–56.6)
Rectal wall V75, %	16.2 (0.0–30.6)
Rectum V50, %	18.6 (2.0–39.4)
Rectum V70, %	9.2 (0.0–34.7)
Bladder wall V47, %	19.0 (6.8–56.7)
Bladder wall V75, %	11.5 (0.0–37.0)
Bladder V50, %	11.8 (2.1–58.5)
Bladder V75, %	5.1 (0.0–35.5)
Femoral head mean dose	
Right, Gy	25.8 (15.4–41.1)
Left, Gy	25.1 (11.3–39.9)
Femoral head max dose	
Right, Gy	35.6 (27.5–60.4)
Left, Gy	35.6 (27.8–63.6)
Penile bulb mean dose, Gy	45.1 (1.8–71.3)
Bowel Max Dose, Gy (RBE)^a^	51.4 (46.0–54.0)

^a^Based on patients receiving whole pelvis treatment

Most patients were treated with two lateral beams. Patients were treated with either uniform scanning (U**N**S) or PBS (our center switched from UNS to PBS in late-2014), with 1- or 2-fields per day. Patients receiving radiation to the pelvic nodes were treated with 2-fields per day. Patients receiving radiation to prostate without pelvic nodes were initially treated two-fields per day and then our center switched to treating one-field per day as the standard in 2015 (alternating between the two lateral beams every other day). This improved efficiency and was not felt to impact treatment quality. For UNS patients, a 0.8–1.2-cm margin was used to account for penumbra. Wax range compensators were designed with an additional range uncertainty of 2.5% + 2 mm added to the distal and proximal ranges, as well as 1–2-cm smearing margins. These were designed using commercially available Xio treatment planning software (Elekta, Stockholm, Sweden).

Dose was verified with an ion chamber measurement performed in water and field shape was verified by comparing the physical shape of the apertures and compensators with the treatment planning system. For PBS delivery, treatment plans were created using the RayStation treatment planning software (RaySearch Laboratories AB, Stockholm, Sweden) with single-field uniform dose optimization. Dose and fluence were measured pre-treatment using the MatrixxPT ion chamber array device (IBA Dosimetry GmbH, Schwarzenbruck, Germany).

A constant relative biological effectiveness (RBE) factor of 1.1 was used to convert physical dose to RBE adjusted dose. In the present study, CGE and RBE adjusted dose are used interchangeably. The robustness of target and organs at risk (OAR) doses was evaluated by computing the plan with ±3% range uncertainty, as well as simulating setup errors by 3-mm isocenter deviations in the anterior/posterior, superior/inferior, and lateral directions. All patients had daily orthogonal kilovoltage x-rays for image guidance prior to treatment with fiducial localization. A digital imaging positioning system was used to determine optimal table shifts along 3 axes to reproduce fiducial localization within 2 mm of the simulation images. Treatment positioning was re-evaluated if more than 5 min passed before beam availability.

## Statistical analysis

Statistical computations were performed with R version 3.1.1 (R Foundation for Statistical Computing, Vienna, Austria). Cumulative incidence of toxicity was estimated using the Kaplan-Meier product limit estimator. Univariate Cox proportional hazards regression was used to assess associations of grade 2 or higher (GR2+) toxicity with potential clinical, treatment-related, and dosimetry variables (listed in Tables 1 and 2). The univariate analysis was considered exploratory and hypothesis-generating, so the *P*-values were not adjusted to account for the number of comparisons. Changes in QOL scores from before and after treatment were analyzed using generalized estimating equation-based linear regression to account for the repeated measurements per patient. Patients who did not have baseline and at least one follow up QOL responses were excluded from that analysis. Throughout the data analysis, two-sided *P* values < 0.05 were considered statistically significant.

## Results

### Patient-reported quality of life

**Fig. 1:**
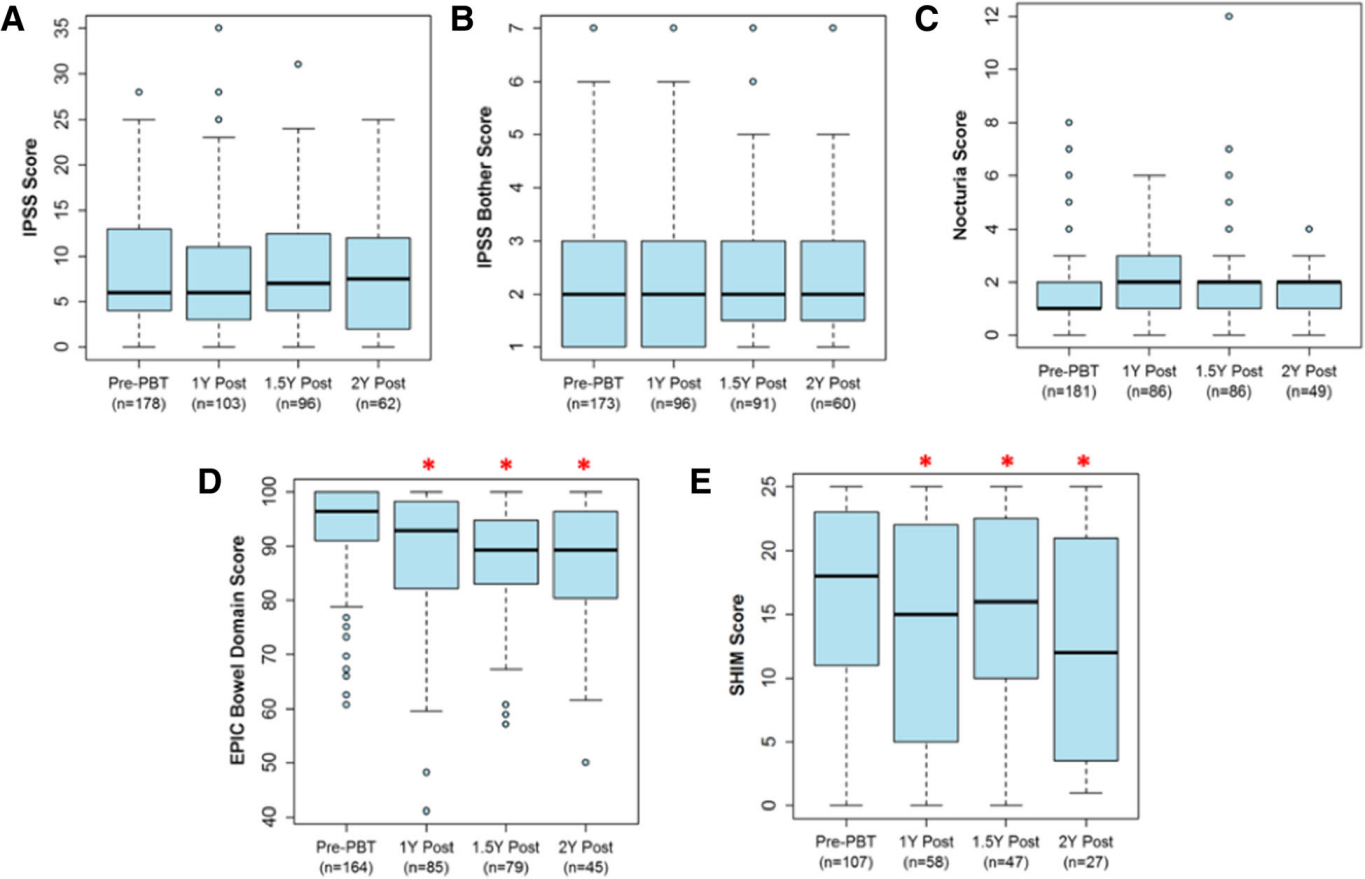
Patient-reported quality-of-life measures. Box-whisker representation of QOL scores at pre-treatment, 1-year, 1.5-year, and 2-years post-treatment (**a**)=IPSS score, (**b**)=IPSS bother score, (**c**)=Nocturia score, (**d**)=EPIC bowel domain score, and (**e**)=SHIM score

Patient reported quality-of-life (QOL) results are detailed in Fig. 1 and Table 3. Urinary function did not change significantly after treatment, as measured by IPSS. Bowel function as measured by EPIC bowel domain scores showed a small decline from 96 at baseline (median) by an average of 5.4 points at 1-year post-treatment (95% CI: 2.5–8.2 points, *p* < 0.001), and then remained stable beyond 1-year (*p* = 0.57). Erectile function as measured by SHIM scores also showed a small decline from 18/25 at baseline (mild ED) by 3.7 points at 1-year post-treatment (95% CI: 1.4–5.9 points, *p* = 0.001). Again, there was no further statistically significant decline beyond 1-year (*p* = 0.25). Treatment with PBS versus UNS did not show a statistically significant difference in toxicity rates, nor did treatment with one-field per day versus two-fields per day.

**Table 3:** Patient-reported QOL results

	Baseline			Follow Up		Mean Change at 1 Year after Treatment			Mean Annualized Change after 1 Year		
QOL Measure	No. of Patients	Median (Range)	Mean ± SD	No. of Patients	No. of Visits	Value	(95% CI)	P-value^a^	Value	(95% CI)	P-value^a^
EPIC Bowel	164	96 (61–100)	93.4 ± 8.5	100	183	−5.4	(−8.2, −2.5)	< 0.001	−1.2	(−5.2, 2.9)	0.57
IPSS	178	6 (0–28)	8.7 ± 6.6	130	241	0.5	(−0.7, 1.8)	0.40	−0.5	(−1.9, 1.0)	0.54
IPSS Bother	173	2 (1–7)	2.5 ± 1.4	125	221	0.3	(−0.1, 0.7)	0.12	−0.2	(−0.6, 0.2)	0.32
Nocturia	181	1 (0–8)	1.8 ± 1.5	123	217	0.3	(−0.0, 0.5)	0.077	−0.2	(−0.5, 0.2)	0.33
SHIM^a^	107	18 (0–25)	16.5 ± 7.1	66	123	−3.7	(−5.9, −1.4)	0.001	1.8	(−1.3, 5.0)	0.25

^a^Only assessed in patients not on androgen deprivation therapy

### Genitourinary (GU) toxicity

One grade 3 (GR3) event was seen within the acute window, an episode of gross hematuria that was found on cystoscopy to be likely related to irritation of a scar from previous TURP. In the acute period (< 90 days from treatment completion), 86 patients (44.8%) required medication for management of urinary irritation (i.e. anti-inflammatory drugs or anti-α1 adrenoceptor blockers), defined as GR2 toxicity, with two patients requiring intermittent self-catheterization. Majority (61/86 patients) reported resolution of symptoms by 6-months post-treatment, and 73/86 had resolution of symptoms by 18 months. Most common symptoms included frequency (50 patients), urgency (44 patients), and dysuria (24).

**Fig. 2:**
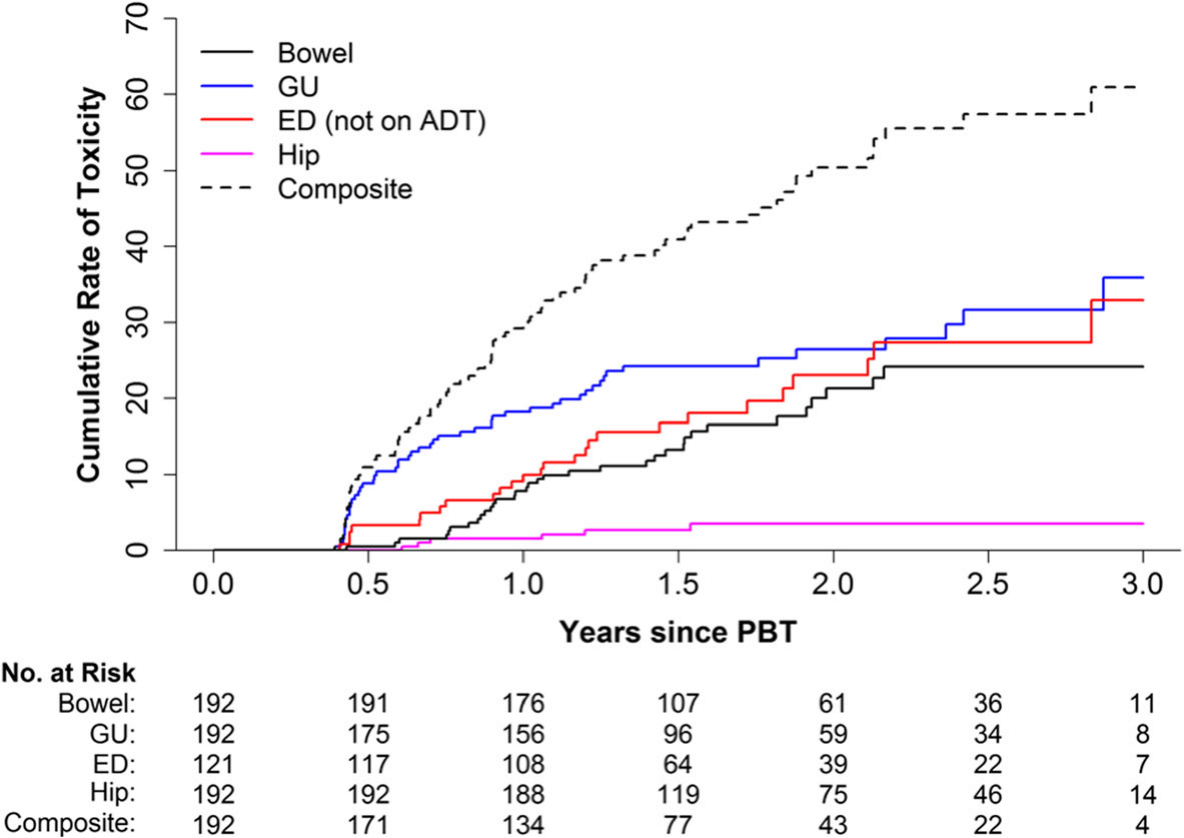
Cumulative actuarial rates for late grade 2+ toxicity (> 90 days post-treatment). Grade 3 toxicity was seen in 5/192 patients, there were no grade 4/5 toxicity. Grade 2 bowel toxicity was mostly transient rectal bleeding managed by enemas/suppositories or laser coagulation. Grade 2 GU toxicity mostly consisted of urinary symptoms managed by α1 adrenoceptor blockers. Grade 2 erectile dysfunction was defined as requiring medications for erectile function. Grade 2 hip pain was pain requiring anti-inflammatory medications

Two patients experienced late GR3 GU toxicity, both radiation cystitis presenting with gross hematuria: one patient required hospitalization for cystoscopy although no intervention was ultimately performed and symptoms resolved; the other patient underwent multiple hyperbaric oxygen treatments with eventual resolution of his symptoms. No patients experienced GR 4 or 5 GU toxicity. Actuarial rate of GR2+ GU toxicity at 2-years was 26.4% (95% CI: 19.4–32.9%), mostly consisting of patients remaining on anti-α1-adrenoceptor blockers for urinary symptoms (Fig. 2). Worsening incontinence was seen in 4 patients, three of whom had some leakage pre-treatment. Of the 51 patients with GR2+ late GU toxicity, 21/51 had resolution of their symptoms within 3–6 months, 24/51 had resolution within 18 months, while the rest remained on anti-α1-adrenoceptor blockers at last follow-up.

Univariate analysis (without adjustment for repeated testing) showed a significant correlation between late GR2+ GU toxicity and increased age (HR = 1.50, *P* = 0.004), higher baseline IPSS score (HR = 1.45, *P* = 0.0046) and higher baseline IPSS bother score (HR = 1.35, *p* = 0.016). No statistically significant associations were found with any other clinical features—including alpha blocker use (*p* = 0.19), ADT (*p* = 0.84), or pre-treatment TURP (*p* = 0.14)—or dosimetric variables. When *p*-values were adjusted for repeated testing, no statistically significant associations were found between toxicity and any variables.

### Gastrointestinal (GI) toxicity

One patient experienced late GR3 toxicity, which was managed with admission for transfusion and resolved after argon plasma coagulation. There were no GR 4 or 5 events. In the acute window, 5 patients reported transient GR2 bowel toxicity, mostly in the form of diarrhea and urgency. Late GR2+ bowel toxicity was seen in 34 patients with an actuarial two-year rate of 21.3% (95% CI: 13.9–28.0%) (Fig. 2). Most observed bowel toxicity was in the form of transient rectal bleeding (32/39 patients) treated with enemas/suppositories or laser coagulation, with the remaining 7 events were due to isolated rectal discomfort or diarrhea. Of the 32 patients who experienced GR2+ rectal bleeding, all events occurred in the late window. Seventeen received medical management with enemas or suppositories, while 15 underwent argon photocoagulation or electrocautery. Bowel toxicity was associated with anticoagulation use (HR = 3.45, *p* = 0.002 without adjustment for repeated testing).

### Erectile dysfunction (ED)

Actuarial two-year rate of late GR2+ ED was 23.0% (95% CI: 13.8–31.3%) (Fig. 2), defined as worsening ED requiring new medical therapies (e.g. sildenafil, tadalafil, vardenafil), with erections firm enough for penetration after medical therapy. Only two patients reported GR3 ED, defined as poor erectile function not responding to oral or injectable medication; both had baseline ED requiring oral medications. On univariate analysis of ED among men not on androgen deprivation therapy, only younger age (HR = 0.6, *p* = 0.022 without adjustment for repeated testing) was associated with decreased late GR2+ toxicity. Mean dose to the penile bulb was not significantly associated with erectile dysfunction.

### Hip toxicity

In the acute window, there were 2 cases of GR2 hip pain requiring anti-inflammatory medication. In the late window, 3 patients reported GR2 hip pain. Three patients reporting maximum GR3 hip pain experienced resolution of symptoms with either cortisone shots to the hip joint, opioid therapy, or eventual hip replacement in one case. The one patient who underwent hip replacement had significant preexisting degenerative changes on pre-treatment imaging. Median time to incidence in those who reported hip pain was 9 months (range 2–29 months). There were no hip fractures. Hip toxicity was not significantly associated with any clinical or dosimetric parameters.

## Discussion

This is one of few contemporary studies that evaluates outcomes and toxicity of PBT for localized prostate cancer with prospectively collected data, including patient reported outcomes. This is the largest series to our knowledge that includes patients treated mostly with PBS, which has dosimetric advantages over older passive scattering proton technology in some scenarios [19]. Although PBT offers known dosimetric advantages over photon radiation, especially in the low to intermediate-dose region (such as volume receiving up to 50 Gy), the clinical impact of this is unclear [7, 20].

In our study population, with median follow up of 1.7 years, we found that rates of GR2+ toxicity outcomes were comparable to other reported data. The low rate of GR3 GU toxicity in this study (5/192 patients) is comparable to other evaluations of PBT, with rates generally < 3% [9]. In the largest prospectively captured data of PBT outcomes for prostate cancer to date, Bryant et al. at the University of Florida found a late GR3 GU actuarial 5-year toxicity rate at of 2.9% [9]. Prior retrospective studies from this group found 1.0% GR3 GU toxicity [13], while other groups have reported a range of 0–2% [15, 17].

As shown in Table 4, reports including the present study consistently show that the risk of grade 3+ GU and GI toxicity is generally low after both PBT and IMRT [9, 13, 15, 17, 21–24]. However, wide variations can be seen in GR2+ toxicity rates with both IMRT and PBT. For example, in two IMRT studies, GU and GI GR2+ toxicity rates reported by Vora et al. were 24.4% and 10.9% respectively, while Spratt et al. reported rates of 8.5% and 2.0% [22, 23]. Multiple factors illustrated in Table 4 may explain this variability and also make true comparison across standalone studies difficult. First, using RTOG versus CTCAE scoring criteria can significantly impact physician-reported toxicity rates, since RTOG criteria depend on subjective assessments of what qualifies as mild (grade 1) versus moderate (grade 2) toxicity, whereas CTCAE criteria require medical or procedural intervention to qualify as grade 2. Second, many studies report observed toxicity rates (numbers of patients experiencing toxicity) with varying lengths of median follow-up [22–24]. This can be addressed by comparing actuarial toxicity rates at specific time intervals, such as 5-years, but these are only inconsistently reported, and at varying time-points themselves [9, 13, 15, 17, 21]. Third, the dose to which patients are treated often varies from study to study. Fourth and finally, treatment planning and patient setup vary from institution to institution.

**Table 4:** Literature review of toxicity data

Study	No. of Patients	Therapy	Median RT Dose Gy or CGE	Median Follow-Up Years	Toxicity Grading Scale	G3+ GU Toxicity	G2+ GU Toxicity	G3+ GI Toxicity	G2+ GI Toxicity
Liauw et al., 2009 [21]	130	IMRT	76	4.4	RTOG	6%^b^	37.0%^b^	5%^b^	14.0%^b^
Spratt et al., 2013 [22]	1002	IMRT	86.4	5.5	CTCAE	2.2%	8.5%	0.7%	2.0%
Vora et al., 2013 [23]	302	IMRT	75.6	7.6	CTCAE	2.6%	24.4%	1%	10.9%
Fang et al., 2015 [24]	94	IMRT	79.2	3.9	CTCAE	0%	18.3%	2.1%	10.8%
Fang et al., 2015 [24]	94	PS PBT	79.2	2.4	CTCAE	2.1%	12.8%	0%	12.8%
Slater et al., 2004 [17]	1255	PS PBT	74	5.3	RTOG	1.0%^a^	–	1.0%^a^	–
Pugh et al., 2013 [15]	291	PS > PBS PBT	76	2.0	RTOG	0%^c^	13.4%^c^	0.3%^c^	9.6%^c^
Mendenhall et al., 2014 [13]	211	PS PBT	78–82	5.2	CTCAE	1.0%^a^	–	0.5%^a^	–
Bryant et al., 2016 [9]	1215	PS PBT	78	5.5	CTCAE	2.9%^a^	–	0.6%^a^	–
Present study, 2017	192	PBS > PS PBT	79.2	1.7	CTCAE	1.0%	26.4%^c^	0.5%	21.3%^c^

*Abbreviations*: *PS* passive scatter, *PBS* pencil beam scanning, *PBT* proton beam therapy, *IMRT* intensity-modulated radiation therapy, *RTOG* radiation therapy oncology group, *CTCAE* common terminology criteria for adverse events, version 4.0

^a^ = 5-year actuarial rate, ^b^ = 4-year actuarial rate, ^c^ = 2-year actuarial rate. No symbol next to a toxicity rate % indicates a rate reported as the number of toxicity events observed over the total number of patients, with varying median follow-up across studies

There is a clear need for studies that can compare PBT and IMRT in a standardized fashion. Although level one evidence comparing IMRT and PBT in patients with prostate cancer is currently being collected in the PARTIQoL and COMPPARE trials (NCT01617161, NCT03561220), results will not be available for years. Thus far, there is conflicting evidence regarding comparative toxicities. In a case-matched comparison of intensity-modulated radiotherapy (IMRT) or PBT based treatment matched for age, GI and GU co-morbidity, and risk group, Fang et al. report that at 2 years there were no differences in GR2 or higher GU or GI toxicities [24]. A Medicare-based comparative study found lower GU toxicity for PBT at 6 months, but no difference at 12 months [18]. Gray et al. compared three dimensional conformal radiotherapy (3D CRT), IMRT, and PBT and found worse acute patient-reported QOL for urinary symptoms for patients treated with 3D CRT but there were no differences by 2 years [12]. A comparison of patient-reported QOL after PBT with patient-reported QOL from the Prostate Cancer Outcomes and Satisfaction (PROSTQA) treatment assessment study of men treated with high-dose photon radiation, showed no differences in any toxicity domain at 2 years [25]. However, a more recent study using private commercial insurance claims reported that PBT was associated with significant reductions in urinary toxicity but increased bowel toxicity three years after treatment [26].

The dosimetric improvement with PBT over IMRT lies in reductions to low and moderate-dose range exposures of normal tissue and not high dose range (70–80 Gy), therefore it is important to qualify the differences that one might expect from PBT in comparison to IMRT [20]. Some of these benefits may be recognized as a decrease in long term toxicity and changes, including the risk of secondary malignancy, and will require much longer follow up with large patient numbers to fully realize [25]. We found no association of toxicity incidence with PBS versus UNS technique in our patient cohort. This is consistent with a previous study that also showed no associations in a cohort treated with mostly passive scatter, and some pencil beam technique [15]. Thus, PBS treatment technique may not clinically affect toxicity rates in prostate cancer treatment, despite potential dosimetric advantages.

Previous retrospective studies have reported excellent biochemical outcomes with PBT [9, 17]. At this time, we did not have enough patient events or follow up time to comment on control rates in this group. We plan to report updated outcomes in the future as patients accrue to the registry and follow up data matures.

Limitations of this study include the patient selection bias for those treated at a single institution. There could also be a selection bias for those patients who sought access to PBT. The follow up period is short, and radiation toxicity is known to develop years after radiotherapy [27]. However, we believe the present study is an important addition to the literature, to assess early outcomes with modern treatment techniques including daily image guidance with intraprostatic fiducials and PBS approach. Strengths include the rigor of prospectively collected toxicity and outcome data, and data that is both patient and physician reported. Future analysis will include continued patient accrual with longer median follow up. The larger patient numbers will allow us to better assess late treatment toxicities and PSA trends amongst patients treated with PBT for prostate cancer at our institution.

## Conclusions

We report toxicity rates in patients treated with contemporary PBT techniques (mostly PBS) at our institution with median 1.7 years of follow up. Grade 3+ toxicities were rare. Patient reported quality-of-life are excellent, with no change in urinary function post-radiation, and small declines in erectile function and bowel QOL by 1-year post-treatment. Larger patient numbers and longer follow up are anticipated to add robustness to our data.

## Abbreviations

CGE: Cobalt gray equivalent
CI: Confidence interval
CT: Computed tomography
CTCAE: Common Terminology Criteria for Adverse Events
CTV: Clinical target volume
ED: Erectile dysfunction
EPIC: Expanded Prostate Cancer Index Composite
GI: Gastrointestinal
GR2: Grade 2
GR2+: Grade 2 or higher
GR3: Grade 3
GR4: Grade 4
GU: Genitourinary
Gy: Gray
HR: Hazard ratio
IMRT: Intensity-modulated radiotherapy
IPSS: International Prostate Symptoms Score
IRB: Institutional review board
MRI: Magnetic resonance imaging
OAR: Organs at risk
PBS: Pencil beam scanning
PBT: Proton beam therapy
PS: Passive scatter
PSA: Prostate specific antigen
PTV: Planning target volume
QOL: Quality of life
RBE: Relative biological effectiveness
SHIM: Sexual Health Inventory for Men
UNS: Uniform scanning
US: Ultrasound
